# Psychosocial impact of skin diseases: A population-based study

**DOI:** 10.1371/journal.pone.0244765

**Published:** 2020-12-31

**Authors:** Yik Weng Yew, Amanda Hui Yu Kuan, Lixia Ge, Chun Wei Yap, Bee Hoon Heng

**Affiliations:** 1 Department of Dermatology, National Skin Centre, Singapore, Singapore; 2 Health Services and Outcomes Research, National Healthcare Group Pte Ltd, Singapore, Singapore; King Faisal University, SAUDI ARABIA

## Abstract

**Background:**

While it is well established that skin disease places significant psychosocial burden on a patient’s wellbeing, its effects have rarely been examined in Asian populations.

**Objective:**

Evaluate the psychosocial burden of skin disease among community-dwelling adults in Singapore.

**Methods:**

This cross-sectional study included 1510 participants interviewed on their history of thirteen skin diseases. The Patient Health Questionnaire (PHQ-9), Lubben Social Network Scale-6 (LSNS-6), University of California Los Angeles (UCLA) Loneliness Scale, and European Quality of Life-5 Dimensions- 5 Level (EQ-5D-5L) were used as measures for depressive symptoms, social isolation, loneliness and quality of life respectively. Multiple linear regression analysis was used to examine the association of skin diseases with each of the four measured outcomes.

**Results:**

Participants with skin diseases reported significantly higher PHQ-9 and UCLA Loneliness scale scores, and lower LSNS-6 and EQ-5D-5L scores when compared to their healthy counterparts. The presence of skin disease was positively associated with depressive symptoms (B = 0.40, SE = 0.11), and negatively associated with quality of life (B = -0.03, SE = 0.01). As disease severity was not evaluated in this study, we were unable to ascertain the associations between disease severity and measured outcomes.

**Conclusion:**

Participants with skin diseases were more likely to have depressive symptoms, social isolation, loneliness and lower quality of life. Unemployed, single and elderly patients were at higher risk of developing depressive symptoms. More emphasis should be placed on the psychosocial aspect of care to reduce the burden of skin disease. Some considerations include monitoring patients for mood-related changes and implementing early psychosocial interventions.

## Introduction

Skin disease contributes 1.79% to the global burden of disease and is the fourth leading cause of non-fatal disease burden [1]. Its socioeconomic implications are well established, with healthcare costs incurred from skin diseases amounting to $75 billion in United States of America [1]. While dermatologists have sought different ways to optimise patient care and reduce the burden of skin disease, the psychosocial aspect of management remains largely overlooked. This is despite the fact that studies have shown that stress can exacerbate skin disease [2]. Patients themselves have also provided feedback that opportunities to express their psychosocial needs are lacking [3]. To optimise patient care, it is important to first understand the psychosocial impact of skin disease. While the psychosocial burden of skin disease is well reported in Western populations, its effects have rarely been examined in Asian populations. Because geographical and ethnic variation in cultural practices and coping mechanisms can augment the psychosocial burden of skin disease, contextualization of evidence is important in disease burden assessment and resource planning. Therefore, we aim to examine the psychosocial burden of skin diseases among a general multi-racial population cohort in Singapore with the intent of increasing awareness on the importance of holistic management.

## Methods

### Study design and participants

This cross-sectional study included 1510 participants who took part in the first-year follow-up survey of the Population Health Survey between November 2016—February 2018. This was a longitudinal health survey on the health of a representative sample of community-dwelling adult population living in the central region of Singapore. The details of the study methodology have been described elsewhere [4]. The eligibility criteria were as follows: (1) Singapore citizens or permanent residents aged ≥ 21 years old; (2) stayed in the selected household for ≥6 months; (3) able to answer the survey questions coherently. Written informed consent was obtained and data was collected via surveyor-administered face-to-face interviews. The ethics approval for this study was obtained from the National Healthcare Group Domain Specific Review Board (Reference Number: 2015/00269), Singapore.

### Skin diseases

The medical history of skin diseases was obtained by asking the participants a specific question: “Have you ever had any of the following skin conditions [a list of 13 skin conditions]?” This included eczema, acne vulgaris, psoriasis, vitiligo, viral warts, scabies, fungal skin infections, chronic urticaria, bacterial skin infections, chronic ulcers or wounds, skin cancers, alopecia areata, and pruritus; and participants were offered an option of “Others” to specify any other skin diseases not listed. This list was modified from the 15 categories of skin diseases included in the Global Burden of Disease Study 2010 [5]. Clinical photographs of each skin disease were used to assist the interview process.

### Instruments and measurements

The outcomes measured in this study were depression, social isolation, loneliness and health-related quality of life (HRQOL).

#### Depressive symptoms

Depressive symptoms were assessed using the 9-item Patient Health Questionnaire (PHQ-9), a well validated and widely used measure of depression. Each item of PHQ-9 is assessed on a 4-point scale (0 = not at all, 1 = several days, 2 = more than half the days, 3 = nearly every day) and the total depressive symptom score for the 9 items ranges from 0 to 27. The scale demonstrated good internal consistency reliability (Cronbach’s alpha = 0.77) in this study.

#### Social isolation

Social isolation was assessed using the Lubben Social Network Scale-6 (LSNS- 6). The LSNS-6 measures the size, closeness and frequency of contacts of a participant’s social network with reference to the level of perceived support they receive from relatives and friends [6]. Each item is scored from 0 to 5, adding up to a total score ranging from 0 to 30, with lower scores indicating increased isolation. The LSNS-6 has demonstrated good internal consistency reliability with Cronbach’s alpha = 0.80 in this study.

#### Loneliness

Loneliness was assessed using the three-item University of California Los Angeles (UCLA) Loneliness Scale [7]. The three items are: “*How often do you feel that you lack companionship*?*”*, “*How often do you feel left out*?*”* and “*How often do you feel isolated from others*?*”*, using a 3-point scale (1 = hardly ever; 2 = some of the time; 3 = often). The scores for each item were added up to produce a score ranging from 3 to 9, with higher scores indicating higher loneliness levels. The internal reliability of the UCLA Loneliness Scale in this study was good, with Cronbach’s alpha = 0.88.

#### Health-related quality of life

HRQOL was assessed using the EQ-5D descriptive system of the European Quality of Life-5 Dimensions-5 Level (EQ-5D-5L) [8]. The descriptive system comprises of five components: mobility, self-care, usual activities, pain/discomfort and anxiety/depression. Each participant can choose to answer from the following options: no problems, slight problems, moderate problems, severe problems or extreme problems. This decision corresponds to a 1-digit number that expresses the level selected for that specific component. The digits for the five components are then combined into a 5-digit number that describes the patient’s health state. The scale demonstrated good internal consistency reliability (Cronbach’s alpha = 0.78) in this study.

### Statistical analysis

Independent-samples t-tests, Chi-square (χ^2^) tests or one-way analysis of variance (ANOVA) were used to assess group differences in socio-demographic characteristics, diagnosis of individual skin diseases, number of chronic diseases, and the measured outcomes, specifically depression, social isolation, loneliness, and HRQOL.

Multiple linear regression analysis was used to examine the association of skin diseases with each of the four measured outcomes. In each regression model, the dependent variable was one of the measured outcomes, and the independent variable was history of any listed skin diseases, adjusted for demographics including age group, gender, ethnicity, marital status, highest education level, employment status, self-reported money sufficiency and diagnosis of any chronic conditions. Descriptive statistics were analyzed using Statistical Package for Social Sciences (SPSS) version 18.0 (SPSS, Inc., Chicago, IL) and multiple linear regression was computed using Stata/SE 12 for Windows. The result was considered significant if p-value was <0.05.

## Results

The average age of the 1510 participants was 54.3 years (standard deviation 16.8 years, range 22–98 years) and 56.2% were women. The majority of participants were Chinese (78.7%), followed by Indians (10.7%) and Malays (7.8%).

The demographics of participants with and without history of any listed skin diseases were compared and described in Table 1. The distributions of age group, marital status, highest education level, employment status and self-reported money sufficiency were significantly different between participants with and without skin diseases. Participants with skin diseases were less likely to be employed, more likely to have financial constraints and alcohol misuse as compared to their healthy counterparts.

**Table 1:** Demographics by history of any listed skin diseases, n (%).

Variable	Total (N = 1,510)	Any skin disease in the list		*p*-value
		No (n = 1,163)	Yes (n = 347)	
Age group				.002
22–39	345 (22.8)	250 (21.5)	95 (27.4)	
40–59	558 (37.0)	444 (38.2)	114 (32.9)	
60–74	419 (27.7)	338 (29.1)	81 (23.3)	
≥ 75	188 (12.5)	131 (11.3)	57 (16.4)	
Gender				.087
Men	662 (43.8)	496 (42.6)	166 (47.8)	
Women	848 (56.2)	667 (57.4)	181 (52.2)	
Marital status				< .001
Single	331 (21.9)	231 (19.9)	100 (28.8)	
Married	926 (61.3)	746 (64.1)	180 (51.9)	
Widowed/Divorced	253 (16.8)	186 (16.0)	67 (19.3)	
Ethnicity				.553
Chinese	1189 (78.7)	916 (78.8)	273 (78.7)	
Malay	118 (7.8)	96 (8.3)	22 (6.3)	
Indian	162 (10.7)	121 (10.4)	41 (11.8)	
Others	41 (2.7)	30 (2.6)	11 (3.2)	
Highest education level				.046
No formal education	160 (10.6)	137 (11.8)	23 (6.6)	
Primary	155 (10.3)	119 (10.2)	36 (10.4)	
Secondary	409 (27.1)	315 (27.1)	94 (27.1)	
Post-secondary & above	786 (52.1)	592 (50.9)	194 (55.9)	
Employment status				.002
Employed	905 (59.9)	703 (60.4)	202 (58.2)	
Unemployed	306 (20.3)	247 (21.2)	59 (17.0)	
Retired	270 (17.9)	198 (17.0)	72 (20.7)	
Permanently unfit for work	29 (1.9)	15 (1.3)	14 (4.0)	
Self-reported money sufficiency				.024
Sufficient	1255 (83.2)	980 (84.4)	275 (79.3)	
Insufficient	253 (16.8)	181 (15.6)	72 (20.7)	
Smoking status				.215
Never smoked	1150 (76.2)	896 (77)	254 (73.2)	
Current smoker	185 (12.3)	141 (12.1)	44 (12.7)	
Former smoker	175 (11.6)	126 (10.8)	49 (14.1)	
Alcohol misuse				.005
No	1168 (77.4)	919 (79.0)	249 (71.8)	
Yes	342 (22.6)	244 (21.0)	98 (28.2)	

The most common skin conditions amongst our participants were eczema (8.8%), followed by bacterial skin infections (7.6%) and fungal skin infections (3.9%). The distributions of the thirteen skin conditions by gender and age are presented in Table 2. Men reported higher rate of fungal skin infections and psoriasis than women. Younger adults (aged 22–39 years old) had higher incidence of acne vulgaris while older adults (aged ≥75 years old) had higher prevalence of bacterial infections and skin cancer. 270 participants (17.9%) complained of pruritus but did not have a specific underlying skin condition identified.

**Table 2:** History of individual skin diseases by gender and age group, n (%).

Skin disease	All	Gender			Age group				
		Men	Women	*p*-value	22–39	40–59	60–74	≥75	*p*-value
Eczema	133 (8.8)	54 (8.2)	79 (9.3)	.430	43 (12.5)	45 (8.1)	27 (6.4)	18 (9.6)	.027
Acne	49 (3.2)	25 (3.8)	24 (2.8)	.303	26 (7.5)	15 (2.7)	7 (1.7)	1 (0.5)	< .001
Psoriasis	21 (1.4)	14 (2.1)	7 (0.8)	.034	2 (0.6)	14 (2.5)	2 (0.5)	3 (1.6)	.025
Vitiligo	7 (0.5)	3 (0.5)	4 (0.5)	.958	1 (0.3)	1 (0.2)	4 (1)	1 (0.5)	.332
Viral warts	21 (1.4)	7 (1.1)	14 (1.7)	.328	5 (1.4)	6 (1.1)	6 (1.4)	4 (2.1)	.762
Scabies	6 (0.4)	3 (0.5)	3 (0.4)	.761	2 (0.6)	2 (0.4)	1 (0.2)	1 (0.5)	.882
Fungal skin infections	59 (3.9)	43 (6.5)	16 (1.9)	< .001	17 (4.9)	20 (3.6)	12 (2.9)	10 (5.3)	.344
Chronic urticaria	16 (1.1)	5 (0.8)	11 (1.3)	.308	4 (1.2)	6 (1.1)	4 (1)	2 (1.1)	.994
Bacterial skin infections	115 (7.6)	60 (9.1)	55 (6.5)	.061	16 (4.6)	37 (6.6)	36 (8.6)	26 (13.8)	.001
Chronic ulcers	24 (1.6)	13 (2)	11 (1.3)	.304	3 (0.9)	3 (0.5)	14 (3.3)	4 (2.1)	.003
Skin cancer	4 (0.3)	2 (0.3)	2 (0.2)	.804	-	1 (0.2)	-	3 (1.6)	.002
Alopecia areata	2 (0.1)	1 (0.2)	1 (0.1)	.861	1 (0.3)	-	-	1 (0.5)	.240
Unspecific symptom of pruritus	270 (17.9)	118 (17.8)	152 (17.9)	.960	57 (16.5)	119 (21.3)	60 (14.3)	34 (18.1)	.036

Participants were interviewed on their comorbidities, with the three most common conditions being hypertension (29.6%), dyslipidaemia (29.1%) and diabetes (14.0%) [Table 3]. Table 3 shows that participants with skin disease reported higher prevalence of chronic conditions including diabetes mellitus, hypertension, dyslipidemia, stroke/transient ischemic attack (TIA), asthma, chronic bronchitis/emphysema/ chronic pulmonary obstructive disease, osteoarthritis/gout/rheumatoid arthritis, depression and anxiety disorder as compared to those without skin disease.

**Table 3:** Diagnosis of self-reported chronic conditions by history of any listed skin disease, n (%).

Self-reported chronic conditions	Any skin disease in the list		*p*-value
	No (n = 1,163)	Yes (n = 347)	
Diabetes	142 (12.2)	69 (19.9)	< .001
Hypertension	325 (27.9)	122 (35.2)	.010
Dyslipidemia	322 (27.7)	117 (33.7)	.030
Heart attack/Ischemic heart disease	19 (1.6)	9 (2.6)	.245
Heart failure	23 (2.0)	13 (3.7)	.058
Stroke/Transient ischemic attack	26 (2.2)	17 (4.9)	.009
Asthma	50 (4.3)	24 (6.9)	.047
Chronic bronchitis /emphysema /Chronic obstructive pulmonary disease	9 (0.8)	8 (2.3)	.018
Chronic kidney disease	12 (1)	9 (2.6)	.029
Cancer	44 (3.8)	13 (3.7)	.975
Osteoarthritis /gout /RA	120 (10.3)	53 (15.3)	.011
Osteoporosis	41 (3.5)	15 (4.3)	.490
Depression	16 (1.4)	11 (3.2)	.027
Anxiety disorder	4 (0.3)	5 (1.4)	.020

Participants with skin diseases reported significantly higher PHQ-9 and UCLA Loneliness scale scores, and lower LSNS-6 and EQ-5D-5L scores compared to those without any skin diseases [Table 4].

**Table 4:** Comparison of PHQ-9, LSNS-6, UCLA loneliness scale and EQ-5D-5L index scores between participants with and without any listed skin diseases, mean±SD.

	Any skin disease in the list		*p*-value
	No (n = 1,163)	Yes (n = 347)	
PHQ-9	0.6 ± 1.7	1.3 ± 2.5	< .001
LSNS-6	16.6 ± 5.9	15.9 ± 6.4	.043
UCLA Loneliness scale	3.3 ± 0.8	3.5 ± 1.2	.002
EQ-5D-5L	0.95 ± 0.12	0.89 ± 0.18	< .001

Linear regression showed that history of skin diseases had a positive correlation with depressive symptoms (B = 0.40, SE = 0.11), and a negative correlation with HRQOL (B = -0.03, SE = 0.01), adjusted for age group, gender, marital status, employment status, highest education level, self-reported money sufficiency, and diagnosis of any chronic diseases [Table 5]. Participants with history of skin diseases were also more likely to feel socially isolated and lonely, although this was not statistically significant.

**Table 5:** Associations between history of any listed skin disease and psychological, social wellbeing and quality of life using multiple linear regressions.

	Depressive Symptoms		Social Engagement		Loneliness		Quality of Life	
	B	SE	B	SE	B	SE	B	SE
History of any skin disease (Ref: no skin disease)	0.398**	0.114	-0.404	0.347	0.106	0.055	-0.033**	0.007
Age group (Ref:22–39)								
40–59	-0.025	0.136	-1.788**	0.415	<0.001	0.066	-0.009	0.009
60–74	-0.363*	0.165	-3.096**	0.501	-0.024	0.079	-0.016	0.010
≥75	-0.213	0.222	-5.023**	0.676	0.055	0.107	-0.092**	0.014
Gender (Ref: Men)	0.159	0.101	0.478	0.308	-0.096*	0.049	-0.015*	0.006
Marital status (Ref: Single)								
Married	-0.225	0.123	1.300**	0.374	-0.197**	0.059	0.015	0.008
Widowed / Divorced	0.001	0.170	0.487	0.516	-0.052*	0.082	0.003	0.011
Employment status (Ref: Employed)								
Unemployed	0.508**	0.131	-1.487**	0.400	0.251**	0.063	-0.006	0.008
Retired	0.440**	0.165	0.458	0.504	0.044	0.080	-0.032**	0.011
Unfit for work	3.372**	0.385	-6.605	1.090	1.429**	0.189	-0.339**	0.023
Highest education (Ref: No formal education)								
Primary	0.093	0.206	-0.069	0.626	0.088	0.099	-0.007	0.013
Secondary	0.080	0.172	0.386	0.524	0.128	0.083	-0.003	0.011
Post-secondary	0.127	0.165	0.144	0.503	0.162	0.080	-0.009	0.011
Self-reported money insufficiency (Ref: sufficient)	0.908**	0.129	-3.244**	0.393	0.466**	0.062	-0.041**	0.008
Diagnosis of any chronic condition (Ref: no chronic conditions)	0.445	0.108	0.248	0.330	0.137**	0.052	-0.036**	0.007

*p < .05

**p < .01

## Discussion

This study explores the impact of skin diseases on psychosocial measures of well-being and HROQL. In this study, participants with history of any skin diseases had a mean EQ-5D index score of 0.89. This is equivalent to people suffering from migraine headaches and is lower than the mean score (0.95) obtained from a large representative sample of community-dwelling residents aged 18 years and above [9]. This highlights the debilitating nature of skin diseases where symptoms such as intractable pruritus, pain, disfigurement can significantly reduce a patient’s quality of life.

Our findings also demonstrated that participants with history of any skin diseases were more likely to have depressive symptoms, social isolation and loneliness. We examine some reasons explaining this observed phenomenon below.

### Financial strain

This study observed that participants with skin diseases were less likely to be employed and more likely to have financial constraints [Table 1]. Across the study population, participants who were not working (including those unemployed, retired or unfit for work) and reported having insufficient money for daily living needs were more likely to have depressive symptoms [Table 5].

Patients with severe skin conditions are more likely to be unfit for work, while those with mild to moderate disease may be able to work but often require time off to attend consultations or take sick leave during flares. A study in Netherlands showed that 64% of patients with atopic dermatitis took sick leave in a year, as compared to 50% of their healthy counterparts [10]. Frequent time away from work can strain employer-employee relationships and result in termination [11], leading to increased financial strain. It is well-established that people with increased financial burden have higher risk of developing depression [12].

### Lack of social relationships

Participants with skin diseases were more likely to be single, including those who were widowed or divorced [Table 1]. Studies have shown that being single increases the risk of developing depression [13]. It is hypothesized that marriage exerts a protective effect on mental well-being, as based on the social support theory of marriage, one’s partner provides not only emotional, but financial and physical support. A study done in United States of America found that adult atopic dermatitis was associated with higher rates of divorce and separation, and postulated that the negative psychosocial impacts of atopic dermatitis such as fatigue, anxiety, depression, sleep disturbances were possible reasons why there was an increased risk of separation and/or divorce [14].

To cope with depression and loneliness, some may turn to maladaptive coping mechanisms such as alcohol. Participants with skin diseases are more likely to have alcohol misuse (28.2%) compared to their healthy counterparts (21%) [Table 1]. This is consistent with a study conducted in the United Kingdom which found that 30.6% of its study subjects with psoriasis had an alcohol misuse disorder compared to 14.3% of the control group [15]. Alcohol misuse not only limits the suitability of various systemic medications used to treat skin conditions, it also further propagates depressive mood symptoms [16].

With a clearer understanding of the psychosocial burden skin diseases have on our patients, we considered a few areas for improvement.

Firstly, more attention should be paid towards patients with a higher risk of developing depressive symptoms, such as those who are not working, single and elderly. Some studies have suggested monitoring patients with a short instrument like PHQ-9 regularly, to detect depression early and refer them to appropriate care accordingly. The early detection of depression is important for patients with any chronic disease, including skin conditions, as studies have shown that patients with depression are three times more likely to be non-adherent to medical treatment as compared to patients without depression [17].

In addition, more emphasis should be placed on psychosocial interventions when managing patients with skin diseases. Existing studies have demonstrated the effectiveness of psychosocial interventions such as cognitive behavioural therapy, both in-person and online [18], mindfulness-based cognitive therapy [19] and structured educational training [20]. These interventions are useful in helping patients positively deal with their condition and promoting adaptation, hence improving patient outcomes [21]. Yet, a local study showed that only 18% of dermatologists report a clear understanding of psychodermatology [22], possibly explaining the scarcity of such interventions in Singapore.

There are several limitations of this study. Firstly, as this was a cross-sectional study, we were unable to clearly ascertain the temporal relationship between skin disease and certain outcomes such as alcoholism. Also, some participants might not have been suffering from skin disease at the point of enrolment, which may have resulted in underestimation of the measured outcomes. Secondly, our study did not evaluate the severity of skin diseases, and hence we were unable to determine the association between severity of skin diseases and measured outcomes. Thirdly, while skin specific quality of life instruments like Dermatology Life Quality Index (DLQI) were not used, EQ-5D has allowed us to compare the impact of skin diseases with other chronic diseases.

Skin diseases have an adverse impact on psychosocial well-being and can lead to more depressive symptoms, social isolation, loneliness and decreased quality of life. The psychological impact of skin diseases is often underestimated compared to that of other chronic diseases. Moving forward, a more holistic approach should be taken to optimise patient care and reduce the burden of skin disease. Some considerations include monitoring patients for mood-related changes regularly and implementing psychosocial interventions early.

## References

[1] Seth D, Cheldize K, Brown D, Freeman EF. Global Burden of Skin Disease: Inequities and Innovations. Curr Dermatol Rep 2017;6(3):204–210. 10.1007/s13671-017-0192-7 29226027 PMC5718374

[2] Foggin E, Cuddy L, Young H. Psychosocial morbidity in skin disease. Br J Hosp Med (Lond) 2017;78(6):C82–C86. 10.12968/hmed.2017.78.6.C82 28614030

[3] Lim DS, Bewley A, Oon HH. Psychological Profile of Patients with Psoriasis. Ann Acad Med Singapore 2018;47(12):516–522. 30636268

[4] Ge L, Yap CW, Ong R, Heng BH. Social isolation, loneliness and their relationships with depressive symptoms: A population-based study. PLoS One 2017;12(8):e0182145 10.1371/journal.pone.0182145 28832594 PMC5568112

[5] Hay RJ, Johns NE, Williams HC, et al The global burden of skin disease in 2010: an analysis of the prevalence and impact of skin conditions. J Invest Dermatol 2014;134(6):1527–1534. 10.1038/jid.2013.446 24166134

[6] Lubben J, Blozik E, Gillmann G, et al Performance of an abbreviated version of the Lubben Social Network Scale among three European community-dwelling older adult populations. The Gerontologist 2006; 46: 503–513. 10.1093/geront/46.4.503 16921004

[7] Hughes ME, Waite LJ, Hawkley LC, Cacioppo JT. A short scale for measuring loneliness in large surveys. Res Aging 2004; 26: 655–672. 10.1177/0164027504268574 18504506 PMC2394670

[8] Herdman M, Gudex C, Lloyd A, et al Development and preliminary testing of the new five-level version of EQ-5D (EQ-5D-5L). Qual Life Res. 2011;20(10):1727–1736. 10.1007/s11136-011-9903-x 21479777 PMC3220807

[9] Abdin E, Subramaniam M, Vaingankar JA, et al Population norms for the EQ-5D index scores using Singapore preference weights. Qual Life Res 2015;24(6):1545–1553. 10.1007/s11136-014-0859-5 25394893

[10] van Os-Medendorp H, Appelman-Noordermeer S, Bruijnzeel-Koomen C, de Bruin-Weller M. Sick Leave and Factors Influencing Sick Leave in Adult Patients with Atopic Dermatitis: A Cross-Sectional Study. J Clin Med 2015;4(4):535–547. 10.3390/jcm4040535 26239345 PMC4470154

[11] Hultin H, Lindholm C, Malfert M, Möller J. Short-term sick leave and future risk of sickness absence and unemployment—the impact of health status. BMC Public Health 2012;12:861 10.1186/1471-2458-12-861 23050983 PMC3508966

[12] Dijkstra-Kersten SM, Biesheuvel-Leliefeld KE, van der Wouden JC, et al Associations of financial strain and income with depressive and anxiety disorders. J Epidemiol Community Health 2015;69(7):660–665. 10.1136/jech-2014-205088 25636322

[13] Bulloch AGM, Williams JVA, Lavorato DH, Patten SB. The depression and marital status relationship is modified by both age and gender. J Affect Disord 2017;223:65–68. 10.1016/j.jad.2017.06.007 28732242

[14] Hua T, Silverberg JI. Atopic dermatitis in US adults: Epidemiology, association with marital status, and atopy. Ann Allergy Asthma Immunol 2018;121(5):622–624. 10.1016/j.anai.2018.07.019 30036584

[15] Al-Jefri K, Newbury-Birch D, Muirhead CR, et al High prevalence of alcohol use disorders in patients with inflammatory skin diseases. Br J Dermatol 2017;177:837–844. 10.1111/bjd.15497 28346655

[16] Boden JM, Fergusson DM. Alcohol and depression. Addiction 2011;106(5):906–914. 10.1111/j.1360-0443.2010.03351.x 21382111

[17] DiMatteo MR, Lepper HS, Croghan TW. Depression is a risk factor for noncompliance with medical treatment: meta-analysis of the effects of anxiety and depression on patient adherence. Arch Intern Med 2000;160(14):2101–2107. 10.1001/archinte.160.14.2101 10904452

[18] van Beugen S, Ferwerda M, Spillekom-van Koulil S, et al Tailored Therapist-Guided Internet-Based Cognitive Behavioral Treatment for Psoriasis: A Randomized Controlled Trial. Psychother Psychosom 2016;85(5):297–307. 10.1159/000447267 27508937

[19] D’Alton P, Kinsella L, Walsh O, et al Mindfulness-Based Interventions for Psoriasis: a Randomized Controlled Trial. Mindfulness 2019;10: 288–300.

[20] Heratizadeh A, Werfel T, Wollenberg A, et al Effects of structured patient education in adults with atopic dermatitis: Multicenter randomized controlled trial. J Allergy Clin Immunol 2017;140(3):845–853. 10.1016/j.jaci.2017.01.029 28242304

[21] Zhang XJ, Wang AP, Shi TY, et al The psychosocial adaptation of patients with skin disease: a scoping review. BMC Public Health 2019;19(1):1404 10.1186/s12889-019-7775-0 31664970 PMC6819547

[22] Chung WL, Ng SS, Koh MJ, et al A review of patients managed at a combined psychodermatology clinic: a Singapore experience. Singapore Med J 2012;53(12):789–793. 23268151

